# Human Parasitic Diseases: A Diagnostic Atlas

**DOI:** 10.3201/eid2608.200270

**Published:** 2020-08

**Authors:** Tonya Crook

**Affiliations:** Penn State Health Milton S. Hershey Medical Center, Hershey, PA, USA

**Keywords:** atlas, parasitology, parasites

Human Parasitic Diseases: A Diagnostic Atlas is a comprehensive and invaluable resource for parasitologists, microbiologists, pathologists, and infectious disease practitioners. Lawrence R. Ash, PhD, and Thomas C. Orihel, PhD, have curated a beautiful photographic series of common and rare parasites shown in tissue, blood, feces, and free-living forms. Organized by phylum, genera, and species, this book provides detailed yet practical assistance in identifying and diagnosing human parasitic diseases. Each section starts with a brief overview of the epidemiology, life cycle, transmission, and clinical manifestations of the parasite, detailed enough to orient the reader to the clinical relevance of the pathogen without distracting from its macroscopic and microscopic diagnostic features. The authors provide up-to-date references of each parasite’s clinical manifestations and diagnostic procedures.

In addition to the beautiful, high-quality photomicrographs, the authors supplement the book with detailed diagrams clarifying the key microscopic diagnostic features. These details enable the reader to differentiate between closely related parasites.

This book offers many unique aspects. First, the authors provide multiple images comparing subtle differences in the appearance of the same parasite, which will reassure anyone who has struggled to identify a blood smear of a parasite that does not quite fit the textbook example. These images emphasize the subtlety of microscopic identification and pattern recognition. Second, this atlas emphasizes the appearance of parasites in histologic findings and tissue. The authors acknowledge the difficulty of making a histologic diagnosis on the basis of fragments of larger parasites or those that have degenerated in tissues. Third, the book contains a 1911 Arthur Looss quote emphasizing the interconnectedness of animal and human parasites and highlighting the need to consider animal pathogens that have rarely infected humans. This perspective is relevant in a world with increasingly immunosuppressed patients and unprecedented levels of travel and global trade. Therefore, this book is a compelling reference volume for pathologists and microbiology or clinical infectious disease training programs.

Particularly useful for today's clinical infectious diseases practitioners is the last section of the book, which covers artifacts for which macroscopic or microscopic appearance could be easily confused even by an experienced pathologist. This section is a helpful reminder of the diagnostic challenges facing clinicians seeing patients who believe they have an infestation but in whom no parasite can be found.

I will certainly use this atlas as a reference and training guide and will most likely browse through its pages before recertification examinations. Any reader with an inclination towards parasitology will appreciate the authors and their colleagues’ fascinating careers in this field.

